# Age-Adjusted Associations Between Routine Systemic Inflammatory Markers and Anti-Müllerian Hormone in Reproductive-Age Women: A Retrospective Cross-Sectional Study

**DOI:** 10.3390/biomedicines14081733

**Published:** 2026-07-31

**Authors:** Mete Hakan Karalök, Bağnu Dündar, Ayhan Parmaksız, Tugba Elgun, Gül Ipek Gündogan, Asiye Gök Yurttaş

**Affiliations:** 1 Department of Obstetrics and Gynecology, Faculty of Medicine, Istanbul Atlas University, Istanbul 34403, Türkiye; hakan.karalok@atlas.edu.tr; 2 Department of Biochemistry, Faculty of Medicine, Istanbul Atlas University, Istanbul 34403, Türkiye; bagnu.dundar@atlas.edu.tr; 3 Department of Biostatistics, Faculty of Medicine, Istanbul Health and Technology University, Istanbul 34015, Türkiye; ayhan.parmaksiz@istun.edu.tr; 4 Department of Medical Biology, Faculty of Medicine, Biruni University, Istanbul 34015, Türkiye; telgun@biruni.edu.tr; 5 Biruni University Research Center (B@MER), Biruni University, Istanbul 34015, Türkiye; ggundogan@biruni.edu.tr; 6 Department of Histology and Embryology, Faculty of Medicine, Biruni University, Istanbul 34015, Türkiye

**Keywords:** anti-Müllerian hormone, ovarian reserve, reproductive age, neutrophil-to-lymphocyte ratio, C-reactive protein, nucleated red blood cells, systemic inflammation, Tobit regression

## Abstract

**Background**: Anti-Müllerian hormone (AMH) is widely used as a marker of ovarian reserve and is strongly influenced by chronological age. Although inflammation has been implicated in ovarian aging and follicular dysfunction, whether routinely measured peripheral inflammatory markers provide additional information regarding AMH concentrations remains unclear. **Objective**: This study aimed to evaluate the age-adjusted associations of C-reactive protein (CRP) and the neutrophil-to-lymphocyte ratio (NLR) with serum AMH concentrations in reproductive-age women. **Methods**: This retrospective cross-sectional study included women aged 18–45 years with available AMH, complete blood count, and CRP measurements. After reapplying the prespecified eligibility criteria and excluding confirmed data-entry or analytical errors, 819 women were included in the final analysis. NLR was calculated from absolute neutrophil and lymphocyte counts. Because CRP and NLR showed right-skewed distributions, Box–Cox transformations were applied. Associations with Box–Cox-transformed AMH were evaluated using an age-adjusted left-censored Tobit regression model. **Results**: The mean age of the participants was 34.07 ± 6.94 years, and the median AMH concentration was 1.07 ng/mL (interquartile range, 0.29–2.50). Chronological age was inversely associated with AMH (β = −0.123, 95% CI: −0.135 to −0.111, *p* < 0.001). After adjustment for age, neither CRP (β = −0.038, 95% CI: −0.085 to 0.009, *p* = 0.114) nor NLR (β = −0.012, 95% CI: −0.222 to 0.198, *p* = 0.912) was independently associated with AMH. **Conclusions**: In this retrospective outpatient cohort, routine peripheral inflammatory markers did not explain additional variation in AMH beyond chronological age. These results do not exclude a potential role for local ovarian inflammation, which may not be adequately captured by peripheral CRP or NLR measurements.

## 1. Introduction

Anti-Müllerian hormone (AMH) is a dimeric glycoprotein produced by granulosa cells of preantral and small antral follicles and is widely used as a biochemical marker of ovarian reserve. Because AMH secretion reflects the pool of growing follicles recruited from the primordial follicle population, serum AMH concentration provides clinically useful information regarding the quantitative aspect of ovarian reserve. Compared with follicle-stimulating hormone, estradiol, and inhibin B, AMH has the advantage of relatively low intra-cycle variability and can therefore be measured independently of menstrual cycle phase in routine clinical practice [1,2,3]. Its age-related decline closely parallels the progressive depletion of the ovarian follicular pool, making chronological age the most consistently recognized determinant of circulating AMH levels [4,5].

Although age remains the dominant predictor of ovarian reserve, AMH concentrations may vary substantially among women of similar age. This interindividual variability suggests that biological factors beyond chronological aging may influence follicular dynamics and granulosa cell function. Metabolic status, endocrine disorders, body composition, oxidative stress, and systemic inflammatory burden have all been proposed as potential contributors to altered ovarian reserve markers [6,7,8]. Among these, inflammation has attracted growing interest because immune signaling is closely involved in several physiological ovarian processes, including follicular development, ovulation, tissue remodeling, and corpus luteum formation. However, sustained or dysregulated inflammatory activity may disrupt this tightly regulated microenvironment and contribute to impaired folliculogenesis, granulosa cell dysfunction, oxidative injury, and accelerated follicular atresia [8,9,10].

Systemic inflammatory status can be assessed using several routinely available hematological and biochemical parameters. The neutrophil-to-lymphocyte ratio (NLR), derived from complete blood count parameters, reflects the balance between innate inflammatory activation and adaptive immune regulation and has been investigated as a low-cost marker of systemic inflammation in various clinical conditions [11]. C-reactive protein (CRP), an acute-phase protein synthesized mainly by hepatocytes in response to pro-inflammatory cytokines, remains one of the most widely used biochemical markers of systemic inflammatory activity [12]. In addition to leukocyte-based indices and CRP, nucleated red blood cells (NRBCs) have recently gained attention as markers of systemic stress, hypoxia, inflammation, and bone marrow response in adult populations, although their role in reproductive medicine remains largely unexplored [13,14].

The potential relationship between systemic inflammation and ovarian reserve is biologically plausible. Pro-inflammatory cytokines, including interleukin-1β, interleukin-6, and tumor necrosis factor-alpha, may influence granulosa cell proliferation, steroidogenesis, oxidative balance, and apoptotic pathways. Oxidative stress, which is closely linked to chronic inflammation, can impair oocyte quality and follicular viability. Moreover, hypoxia-related pathways and inflammatory activation may interact within the ovarian microenvironment, potentially affecting follicular development and AMH secretion [8,9,10,15]. Nevertheless, available clinical evidence remains inconsistent. Some studies have suggested inverse associations between inflammatory markers and ovarian reserve parameters in selected populations, such as women with infertility, polycystic ovary syndrome, endometriosis, or diminished ovarian reserve. However, whether routinely measured systemic inflammatory markers are independently associated with AMH levels in a broader reproductive-age outpatient population remains uncertain [6,7,16].

A key methodological challenge in evaluating AMH is its highly skewed distribution, strong age dependency, and lower-limit censoring due to assay detection thresholds. Standard linear modeling approaches may therefore provide biased or unstable estimates when these distributional characteristics are not appropriately addressed. In addition, inflammatory indices such as NLR and CRP are themselves frequently right-skewed and may require suitable transformation or robust modeling strategies. These considerations are particularly important when attempting to determine whether inflammatory markers contribute independently to AMH variability beyond the well-established effect of chronological age.

Routine inflammatory markers are attractive in clinical practice because they are inexpensive and widely available. However, CRP and NLR are nonspecific peripheral indices that may not directly represent inflammatory activity within the ovarian stroma, granulosa cells, or follicular fluid. Determining whether these markers provide information beyond chronological age is therefore clinically relevant, particularly because they should not be interpreted as surrogate markers of ovarian reserve without evidence of an independent association with AMH.

Therefore, this retrospective cross-sectional study aimed to evaluate whether routinely available peripheral inflammatory markers, particularly CRP and NLR, were associated with serum AMH concentrations after accounting for chronological age in reproductive-age women attending an outpatient gynecology clinic. A secondary objective was to determine whether the addition of these inflammatory markers provided information beyond an age-based model.

## 2. Materials and Methods

### 2.1. Study Design and Setting

This retrospective, observational, cross-sectional study was conducted at Atlas University Hospital. The study was designed and reported in accordance with the general principles of the Strengthening the Reporting of Observational Studies in Epidemiology (STROBE) statement for observational studies [17]. Data were obtained from the Hospital Information Management System (HIMS) and the Laboratory Information System (LIS) for women who attended the outpatient Obstetrics and Gynecology clinic between 1 January 2020 and 31 December 2025.

The study protocol was approved by the relevant institutional ethics committee before data extraction. Because of the retrospective design, no additional diagnostic or interventional procedures were performed. All data were anonymized before analysis, and patient identifiers were replaced with unique numerical codes to ensure confidentiality in accordance with applicable data protection regulations.

Clinical and laboratory records were generated during routine patient care between 1 January 2020 and 31 December 2025. Following ethics committee approval in June 2026, de-identified data were extracted from the hospital information systems specifically for the present study. No research-specific data extraction or statistical analysis was conducted before ethical approval.

### 2.2. Study Population

The study population consisted of women of reproductive age who had undergone serum anti-Müllerian hormone (AMH) measurement as part of routine clinical evaluation during the study period. AMH is widely used as a marker of ovarian reserve because it is secreted by granulosa cells of preantral and small antral follicles and shows relatively low intra-cycle variability compared with several other endocrine markers [1,2,3]. A consecutive sampling strategy was used, and all patients who met the eligibility criteria and had complete laboratory data were included in the final analytical cohort.

An a priori sample size calculation was performed based on the assumption of a moderate correlation between AMH and inflammatory markers (r = 0.25), with a two-tailed alpha level of 0.05 and 80% statistical power. The minimum required sample size was estimated to be approximately 124 participants. To improve the precision of estimates and maximize statistical power, all eligible patients with complete records during the study period were included.

### 2.3. Eligibility Criteria

Women were eligible for inclusion if they met the following criteria:

- Age was calculated at the date of AMH measurement using the date of birth recorded in the hospital information system. Only women aged 18.00–45.99 years at the time of AMH testing were included.
- Female sex.
- Attendance at the outpatient Obstetrics and Gynecology clinic during the study period.
- Availability of serum AMH measurement performed as part of routine clinical assessment.
- Availability of complete blood count and CRP measurements in the electronic medical records.

Patients were excluded if they had any of the following:

- Confirmed or suspected pregnancy at the time of blood sampling.
- Known or previously diagnosed hematological or solid-organ malignancy.
- Acute infectious disease or active inflammatory condition at the time of sampling.
- Documented autoimmune disease, including systemic lupus erythematosus, rheumatoid arthritis, or clinically active autoimmune thyroiditis.
- Chronic corticosteroid use or immunosuppressive therapy within the preceding three months.
- Missing or incomplete clinical or laboratory data in the HIMS or LIS records.

### 2.4. Data Collection and Variables

Data were extracted retrospectively from electronic medical records using a standardized data collection form developed by the research team. No direct patient contact was established, and no surveys, interviews, or questionnaires were conducted.

The following variables were recorded for each participant: age in years, serum AMH concentration in ng/mL, serum C-reactive protein (CRP) level in mg/L, and complete blood count parameters, including absolute neutrophil count, absolute lymphocyte count, platelet count, and nucleated red blood cell count (NRBC). Complete blood count parameters were expressed as ×10^3^/μL where applicable, while NRBC was recorded as cells per 100 white blood cells. Only complete blood count and CRP measurements obtained on the same date as the AMH measurement were included.

Serum AMH concentrations were measured using the VIDAS^®^ AMH assay (bioMérieux SA, Marcy-l’Étoile, France), based on the ELFA (Enzyme-Linked Fluorescent Assay) method, on a VIDAS 3 analyzer (bioMérieux). The analytical measurement range of the AMH assay was 0.02–9.00 ng/mL. Results at the lower reporting limit were treated as left-censored observations at 0.02 ng/mL in the Tobit regression analyses. Results reported below this limit were recorded as 0.02 ng/mL and treated as left-censored observations in the regression analysis. CRP was measured using a turbidimetric method on an Abbott Architect c8000 analyzer (Abbott Laboratories, Abbott Park, IL, USA), and complete blood count parameters were measured using a Sysmex XN-1000 automated hematology analyzer (Sysmex Corporation, Kobe, Japan).

All laboratory measurements had been performed as part of routine clinical care using standardized laboratory procedures. NRBC values were obtained from automated hematology analyzer differentials, which identify nucleated erythrocytes as a separate parameter during white blood cell differential analysis. NRBCs are immature erythroid precursors that are generally absent from the peripheral blood of healthy adults and may appear under conditions associated with systemic stress, inflammation, hypoxia, or bone marrow activation [13,14].

The neutrophil-to-lymphocyte ratio (NLR) was calculated by dividing the absolute neutrophil count by the absolute lymphocyte count. NLR was evaluated as a readily available hematological index reflecting the balance between neutrophil-mediated inflammatory activity and lymphocyte-associated immune regulation [11]. CRP was evaluated as a biochemical marker of systemic inflammation because it is an established acute-phase protein induced by pro-inflammatory cytokine signaling [12]. NRBC was initially considered as a hematological indicator of systemic stress and inflammation; however, because of its highly sparse distribution and near-zero variance in this outpatient population, its suitability for multivariable modeling was evaluated before final model construction.

Information regarding BMI, smoking status, menstrual cycle phase, PCOS, endometriosis, metabolic disorders, thyroid function, vitamin D status, medication use, and reproductive history was not consistently recorded in the retrospective database and could therefore not be included in the primary multivariable analysis.

### 2.5. Statistical Analysis

Statistical analyses were performed using R version 4.5.1 (R Foundation for Statistical Computing, Vienna, Austria). Continuous variables were summarized as mean ± standard deviation and median with interquartile range, as appropriate. Distributional characteristics were examined before modeling. Because AMH and the inflammatory variables showed right-skewed distributions and nonlinear relationships, Box–Cox power transformations were applied, with lambda parameters estimated by maximum likelihood [18,19]. Age was not transformed. Pearson correlation coefficients were calculated for the bivariate analyses. Because observations at the lower reporting limit of the AMH assay were left-censored, a left-censored Tobit regression model was fitted with Box–Cox-transformed AMH as the dependent variable [20]. The censoring threshold was defined as the Box–Cox-transformed value corresponding to the verified assay lower reporting limit. Alternative models were compared using variance inflation factors, log-likelihood, AIC, BIC, and back-transformed predictive performance. Regression coefficients were reported with 95% confidence intervals and two-sided *p* values. A *p* value below 0.05 was considered statistically significant.

## 3. Results

### 3.1. Participant Selection and Characteristics

Before multivariable modeling, the dataset was examined for missing values, distributional properties, influential observations, and high-leverage data points. During diagnostic assessment, an extreme value was identified in the nucleated red blood cell (NRBC) parameter and was excluded from correlation-based analyses. AMH values within the verified analytical measurement range were retained unless a documented data-entry error, unit error, duplicate record, or analytical error was identified.

After outlier management, the final analytical cohort included 848 women. NRBC showed a highly sparse distribution, with 819 of 848 participants (96.6%) having undetectable values. Because of this near-zero variance, NRBC was not included in the multivariable regression models. Instead, NRBC was described descriptively and interpreted cautiously as a limited exploratory variable in this outpatient cohort.

A total of 848 records were identified. After application of the prespecified age criterion, 12 records from women younger than 18 years and 17 records from women older than 45 years were excluded. Following the remaining eligibility and data-quality checks, the final analytical cohort included 819 women. In the final cohort, NRBC values were undetectable in [NRBC n] of 819 participants ([NRBC %]). All descriptive, bivariate, multivariable, and sensitivity analyses were performed using this final cohort. Owing to this highly sparse distribution and near-zero variance, NRBC was not included in the multivariable regression models and was retained only as a descriptive exploratory variable.

### 3.2. Descriptive and Bivariate Analyses

Descriptive analyses showed that AMH, CRP, and NLR had right-skewed distributions. Exploratory bivariate analyses also demonstrated a non-linear relationship between chronological age and AMH. Therefore, Box–Cox power transformations were applied to laboratory variables to reduce skewness, improve variance stability, and better approximate linear relationships. Age was not transformed.

The optimal lambda values estimated by maximum likelihood were as follows: 0.2697 for AMH, 0.0626 for CRP, −0.0253 for neutrophil count, 0.7191 for lymphocyte count, and −0.5053 for NLR. These transformations improved the distributional characteristics of the variables and reduced the non-linearity observed between age and AMH. After transformation, the relationship between age and AMH was more suitable for linear and censored regression modeling (Table 1).

### 3.3. Age-Adjusted Associations with AMH

Four candidate Tobit regression models were evaluated. Model 1 included age, CRP_BC, Neutrophil_BC, and Lymphocyte_BC; Model 2 included age, CRP_BC, and NLR_BC; Model 3 included age, CRP_BC, Neutrophil_BC, Lymphocyte_BC, and NLR_BC; and Model 4 included age, CRP_BC, and Neutrophil_BC. Model 3 was fitted only as a diagnostic model to assess the multicollinearity arising from the simultaneous inclusion of neutrophil count, lymphocyte count, and their derived ratio, NLR. Because this model showed problematic multicollinearity, it was not considered for substantive interpretation. Among Models 1, 2, and 4, Model 2 yielded the lowest AIC and BIC values and was therefore selected as the final multivariable model (Table 2).

### 3.4. Assessment of Left-Censoring and Model Selection

**Figure 1:**
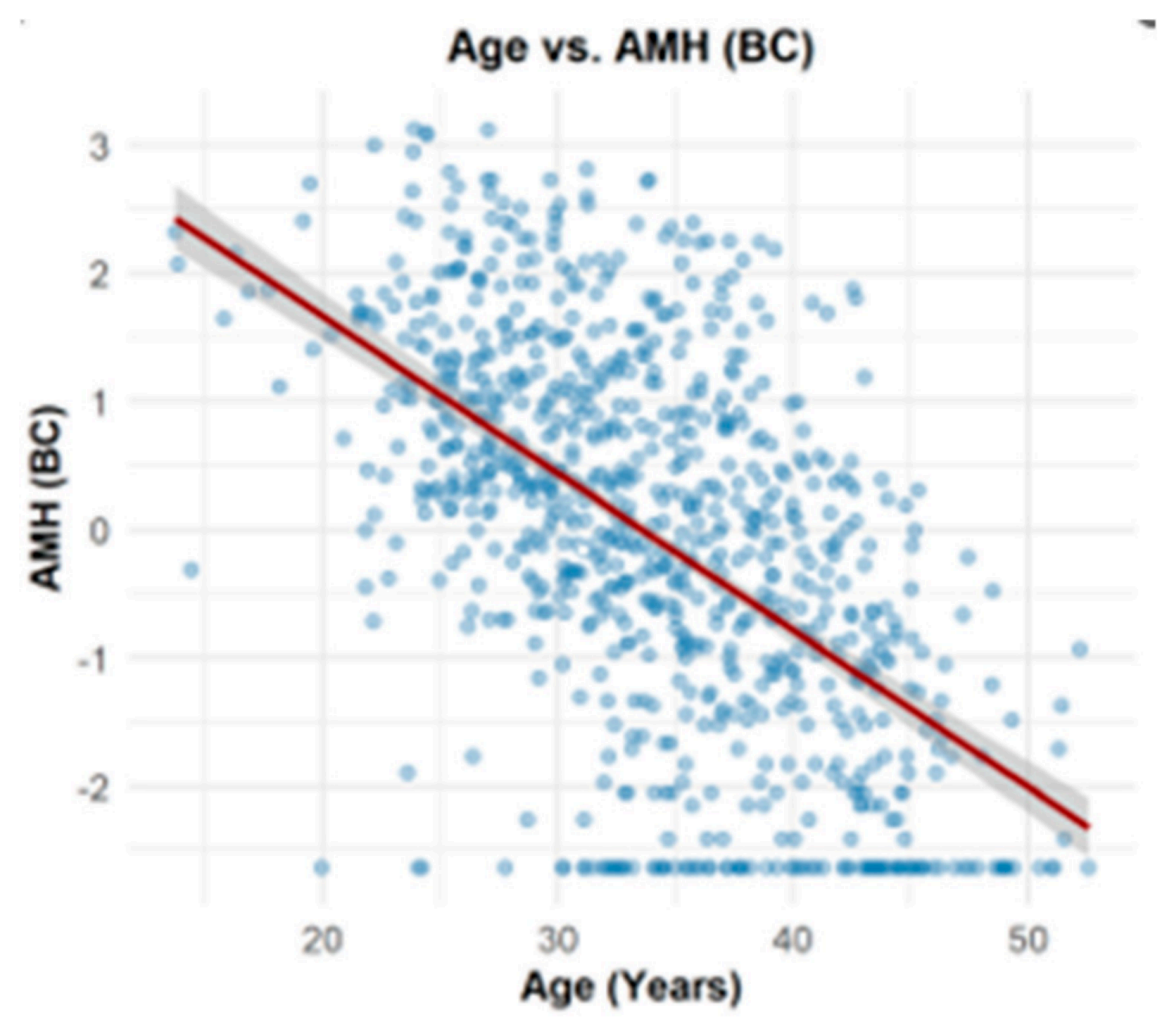
Association between chronological age and Box–Cox-transformed serum AMH concentration.

Post-transformation scatter plots demonstrated a visible floor effect in the dependent variable, corresponding to the lower limit of detection (LOD) of the AMH assay. This pattern indicated that AMH values were left-censored at the lower measurement boundary rather than being fully continuous across the entire range. In addition, clustering of low CRP values was observed, most likely reflecting assay-related measurement limits. Because ordinary least squares regression may produce biased estimates when the dependent variable is censored at a known boundary, Tobit regression was used for multivariable modeling. The left-censoring threshold was defined as the Box–Cox transformed value corresponding to the empirical lower AMH limit of 0.02 ng/mL, which was 2.41688 (Figure 1).

To identify the most appropriate multivariable model, four alternative Tobit regression models were constructed and compared using variance inflation factors (VIFs) to assess multicollinearity and Akaike information criterion (AIC) and Bayesian information criterion (BIC) values to evaluate model fit (Table 2).

Model 3, which included neutrophil count, lymphocyte count, and NLR simultaneously, showed evidence of multicollinearity, with VIF values of 6.919 for Neutrophil_BC and 6.914 for Lymphocyte_BC. Therefore, this model was not selected for final interpretation.

Among the remaining models, Model 2 and Model 4 showed comparable predictive performance after back-transformation to the original AMH scale, with identical original-scale R^2^ values and RMSE estimates. However, Model 2 yielded the lowest AIC and BIC values and included NLR as a clinically interpretable composite inflammatory index. Therefore, the ratio-based model including age, CRP_BC, and NLR_BC was selected as the final multivariable Tobit regression model.

### 3.5. Validation and Clinical Parameter Interpretation

The final ratio-based Tobit model (Model 2) provided a significantly better fit than the intercept-only model (likelihood ratio test: χ^2^ = 420.6, *p* < 0.001). The Cox and Snell and Nagelkerke pseudo-R^2^ values were 0.3315 and 0.3406, respectively. These values are presented as descriptive indicators of model performance and should not be interpreted in the same manner as the R^2^ obtained from ordinary least squares regression. The maximum likelihood parameter estimates for the final model are presented in Table 3.

In the final Tobit regression model, chronological age was inversely associated with Box–Cox-transformed AMH (β = −0.123, 95% CI: −0.135 to −0.111, *p* < 0.001). After adjustment for age, neither CRP_BC (β = −0.038, 95% CI: −0.085 to 0.009, *p* = 0.114) nor NLR_BC (β = −0.012, 95% CI: −0.222 to 0.198, *p* = 0.912) was independently associated with AMH. Because the coefficients are reported on the Box–Cox-transformed scale, they should not be interpreted as direct changes in AMH concentration in ng/mL.

### 3.6. Methodological Validation on the True Clinical Scale

To evaluate the performance of the final Box–Cox Tobit model, predictions from alternative modeling approaches were back-transformed to the original, non-transformed AMH scale in ng/mL. Model performance was then compared using original-scale R^2^, RMSE, and MAE values, as summarized in Table 4.

When predictions were back-transformed to the original AMH scale, the log-Tobit model showed lower predictive performance than the Box–Cox based approaches, with an original-scale R^2^ of 0.1477, RMSE of 2.0081 ng/mL, and MAE of 1.2043 ng/mL. In contrast, both Box–Cox based models yielded higher original-scale explanatory performance and lower prediction errors. The Box–Cox OLS model and the final Box–Cox Tobit model showed comparable predictive metrics after back-transformation, with an original-scale R^2^ of 0.2288, RMSE of 1.7439 ng/mL, and MAE of 1.1114 ng/mL. However, the Box–Cox OLS model does not account for the left-censoring observed at the lower limit of AMH detection. Therefore, although its predictive performance was similar, it was considered less appropriate for the structure of the present dataset. The final Box–Cox Tobit model was retained as the preferred modeling approach because it accounted for both the skewed distribution of AMH and the left-censoring caused by the assay’s lower limit of detection. This approach provided clinically interpretable predictions on the original AMH scale while maintaining an appropriate statistical framework for censored outcome data.

## 4. Discussion

In this retrospective cross-sectional study of reproductive-age women, chronological age showed a strong inverse association with serum AMH concentrations. After adjustment for age, routine peripheral inflammatory markers, including CRP and NLR, were not independently associated with AMH. These findings indicate that CRP and NLR did not provide additional information beyond age in the present outpatient cohort. However, they should not be interpreted as evidence that inflammation is unrelated to ovarian aging or follicular function.

AMH is secreted by granulosa cells of preantral and small antral follicles and is widely accepted as a clinically useful marker of ovarian reserve. Its decline with increasing age reflects the progressive depletion of the follicular pool and remains one of the most consistent findings in reproductive endocrinology [1,2,3,4]. The strong inverse association between age and AMH observed in the present study is therefore biologically expected and supports the internal validity of the dataset. Importantly, the strength of this association increased after Box–Cox transformation, suggesting that appropriate handling of AMH skewness and non-linear age-related decline improves the statistical representation of the age–AMH relationship.

The potential role of systemic inflammation in ovarian reserve has gained increasing attention in recent years. Chronic low-grade inflammation has been proposed as one of the mechanisms contributing to ovarian aging, alongside oxidative stress, mitochondrial dysfunction, altered immune cell activity, stromal remodeling, and ovarian fibrosis [21,22]. Within the ovarian microenvironment, inflammatory mediators may influence follicular activation, granulosa cell function, steroidogenesis, oocyte quality, and follicular atresia. Zeng et al. emphasized that chronic inflammation may accelerate ovarian aging through inflammasome activation, cytokine signaling, oxidative injury, and tissue remodeling pathways [21]. Similarly, recent reviews on reproductive aging have highlighted the accumulation of immune cells, inflammatory signaling, and fibrosis as key components of age-related ovarian functional decline [22,23]. These biological observations provide a plausible rationale for investigating whether systemic inflammatory markers are associated with AMH levels in clinical populations.

Despite this biological plausibility, the present study did not demonstrate an independent association between CRP or NLR and AMH after adjustment for age. This finding should not be interpreted as evidence that inflammation has no role in ovarian aging or follicular function. Rather, the absence of an independent association may reflect important biological differences between systemic and local ovarian inflammation. CRP is a nonspecific hepatic acute-phase reactant, whereas NLR broadly reflects the balance between circulating neutrophils and lymphocytes. In contrast, inflammatory signaling within the ovary may be compartmentalized within the ovarian stroma, granulosa cells, theca cells, and follicular fluid. Local cytokine activity may therefore influence follicular activation, steroidogenesis, granulosa cell survival, and follicular atresia without producing measurable changes in routine peripheral blood markers. Furthermore, the systemic inflammatory burden in the present cohort may have been relatively limited because women with acute infection, active inflammatory disease, autoimmune disorders, malignancy, or immunosuppressive treatment were excluded [8,9,10,21,22,23].

The current findings differ from some recent studies conducted in infertility or assisted reproduction settings. In a large retrospective cohort of women undergoing ART, Yao et al. reported that higher systemic inflammatory indices, including SII, PLR, PPN, and NLR, were associated with lower AMH, lower antral follicle count, and fewer retrieved oocytes after multivariable adjustment [24]. Similarly, studies in fertility clinic populations have reported differences in NLR, PLR, and related hematological indices between women with normal and diminished ovarian reserve [25]. These discrepancies may be explained by differences in study populations, clinical context, timing of blood sampling, inclusion criteria, and covariate adjustment. Women undergoing ART or evaluated for infertility may have a higher burden of reproductive pathology, endocrine dysfunction, endometriosis, PCOS, metabolic abnormalities, or subclinical inflammation compared with a broader outpatient gynecology population. Therefore, inflammatory markers may show stronger associations with ovarian reserve in selected infertility cohorts than in general reproductive-age outpatient samples.

CRP is a classical acute-phase reactant and one of the most commonly used biochemical markers of systemic inflammation. Although CRP has been linked to adverse reproductive and metabolic profiles in some studies, its relationship with AMH is not uniform across populations. In the present study, CRP showed no significant independent association with AMH after age adjustment. This may reflect the non-specific nature of CRP, which is influenced by adiposity, metabolic status, recent infection, medications, smoking, and other systemic conditions. Because BMI, smoking status, vitamin D level, insulin resistance, thyroid function, and detailed menstrual or endocrine parameters were not available in the retrospective dataset, residual confounding cannot be excluded. Nevertheless, the absence of an independent CRP–AMH association suggests that isolated CRP measurement may have limited clinical utility for interpreting AMH in women without overt inflammatory disease.

NLR was also not independently associated with AMH in the final model. NLR is an accessible hematological index reflecting the balance between neutrophil-mediated innate immune activation and lymphocyte-related immune regulation. It has been investigated in several reproductive conditions, including infertility, PCOS, endometriosis, ovarian stimulation response, and pregnancy-related disorders. However, NLR is also a non-specific marker and may vary according to stress, infection, metabolic status, medications, and timing of blood sampling. The lack of association in the present study may indicate that NLR alone does not adequately represent the complex immune and oxidative changes involved in ovarian aging. It is also possible that composite indices such as SII or PLR, or direct cytokine measurements such as IL-6, TNF-α, and IL-1β, may be more informative than NLR alone in selected clinical settings.

More specific inflammatory biomarkers may be more informative in selected populations. Circulating or follicular-fluid concentrations of IL-6, IL-1β, TNF-α, and other cytokines, together with oxidative stress and antioxidant markers, may provide a more direct assessment of inflammation-related ovarian changes. However, these biomarkers were not available in the present retrospective dataset and cannot be inferred from routine CRP or NLR measurements.

The inclusion of NRBC as an exploratory hematological marker represents a novel aspect of this study. NRBCs are immature erythroid precursors that are generally absent from the peripheral blood of healthy adults. Their appearance in peripheral circulation has been associated with systemic stress, hypoxia, severe inflammation, sepsis, and bone marrow activation [26,27,28]. Mechanistically, NRBC release may reflect the combined effects of inflammatory cytokines, hypoxic signaling, and stress erythropoiesis. Because hypoxia and inflammation are also implicated in ovarian dysfunction and follicular atresia, NRBC was considered a potentially interesting marker of systemic stress in relation to ovarian reserve. However, in the present cohort, 96.6% of participants had undetectable NRBC values, resulting in near-zero variance. Therefore, NRBC could not be meaningfully evaluated as an independent predictor of AMH in multivariable models. This finding suggests that NRBC may not be a useful marker in relatively stable outpatient reproductive-age populations, although it may still warrant exploration in cohorts with greater systemic inflammatory or hypoxic burden.

Inflammation and oxidative stress are closely interconnected within the follicular microenvironment. Excessive reactive oxygen species may impair mitochondrial function, granulosa cell viability, oocyte competence, and steroidogenesis, whereas inadequate antioxidant capacity may increase susceptibility to follicular atresia. Studies examining follicular fluid have reported alterations in local inflammatory mediators and oxidative stress markers among women with diminished ovarian reserve or impaired reproductive outcomes. These more proximal biomarkers may reflect ovarian biological processes more directly than CRP or NLR measured in peripheral blood. Thus, the negative findings of the present study may indicate limited sensitivity of routine systemic markers rather than the absence of inflammatory or oxidative mechanisms in ovarian aging [10,21,22,23,28].

The methodological approach used in the present study is another important aspect of interpretation. AMH values are typically right-skewed and may show a lower-boundary effect because of assay detection limits. Standard linear regression may therefore be suboptimal if skewness and censoring are not addressed. In this study, Box–Cox transformation improved the distributional characteristics of AMH and inflammatory variables, while Tobit regression accounted for left-censoring at the AMH assay’s lower limit of detection. This approach provided a more appropriate statistical framework for evaluating independent predictors of AMH. Importantly, after applying this modeling strategy, age remained the only statistically significant determinant of AMH, whereas CRP and NLR did not contribute independently.

From a clinical perspective, the present findings do not support the use of isolated CRP or NLR measurements as substitutes for AMH or established ovarian reserve assessment. Their absence of an age-adjusted association in this cohort suggests that routine interpretation of AMH should not be modified solely on the basis of these nonspecific inflammatory markers. Nevertheless, CRP and NLR may remain clinically relevant for other indications and may show different associations in women with PCOS, endometriosis, infertility, metabolic disease, or active inflammatory conditions.

The present study has several strengths. First, it included a relatively large sample of reproductive-age women from routine gynecology outpatient practice. Second, patients with major conditions that could strongly confound inflammatory markers, including acute infection, active inflammatory disease, malignancy, autoimmune disease, corticosteroid use, and immunosuppressive therapy, were excluded. Third, the study evaluated both biochemical and hematological inflammatory markers, including CRP, NLR, and the exploratory marker NRBC. Fourth, the statistical analysis accounted for skewed AMH distribution, non-linear age-related decline, multicollinearity, and left-censoring due to assay detection limits.

Several limitations should be considered. First, the retrospective cross-sectional design precludes causal inference and does not allow assessment of temporal relationships between inflammatory status and AMH concentrations. Second, important clinical, metabolic, and lifestyle variables, including BMI, smoking status, PCOS, endometriosis, metabolic disorders, thyroid disease, vitamin D status, hormonal medication use, menstrual cycle characteristics, and reproductive history, were not consistently available in the retrospective database. Because several of these factors may influence both inflammatory markers and AMH concentrations, residual confounding remains possible. Third, PCOS status could not be reliably identified in all participants. High AMH concentrations were retained in the primary analysis because they may represent biologically valid findings, particularly in women with PCOS or other hyperandrogenic phenotypes; however, subgroup-specific associations could not be evaluated. Fourth, CRP and NLR are nonspecific peripheral inflammatory markers and may not adequately reflect inflammatory activity within the ovarian stroma, granulosa cells, or follicular fluid. Fifth, the timing of inflammatory measurements in relation to transient illness, physiological stress, medication use, and menstrual cycle phase could not be fully standardized. Finally, the single-center design may limit the generalizability of the findings to other clinical populations.

## 5. Conclusions

In this retrospective cross-sectional study, chronological age was strongly associated with serum AMH concentrations. After adjustment for age, CRP and NLR did not show independent associations with AMH and did not provide substantial additional information beyond an age-based model in this outpatient cohort.

These findings apply specifically to routine peripheral inflammatory markers and should not be interpreted as evidence that inflammation has no role in ovarian aging or follicular function. Local ovarian inflammation, oxidative stress, specific cytokine profiles, and clinical conditions such as PCOS or endometriosis may not be adequately represented by CRP or NLR. The results therefore do not support using isolated CRP or NLR measurements as surrogate indicators of ovarian reserve.

## Figures and Tables

**Table 1:** Association between chronological age and serum AMH concentration in the final analytical cohort of women aged 18.00–45.99 years.

*Variable*	*Mean ± SD*	*Median (Q1–Q3)*	*Min–Max*	*Correlation Coefficient (r)*	*p Value*
*Raw variables*					
*Age, years*	34.07 ± 6.94	33.84 (28.92–39.11)	18.00–45.99	−0.495	<0.001
*AMH, ng/mL*	1.72 ± 1.90	1.07 (0.29–2.50)	0.02–9.00	Reference	—
*CRP, mg/L*	10.29 ± 23.50	2.73 (2.00–9.60)	0.10–354.30	−0.024	0.482
*Neutrophil count, ×10^3^/μL*	4.66 ± 2.18	4.08 (3.19–5.75)	0.95–14.50	0.047	0.175
*Lymphocyte count, ×10^3^/μL*	2.13 ± 0.79	2.10 (1.64–2.60)	0.18–5.97	0.044	0.204
*NLR*	2.85 ± 3.46	1.90 (1.44–2.67)	0.40–42.04	0.020	0.553
*NRBC, cells/100 WBC*	0.01 ± 0.04	0.00 (0.00–0.00)	0.00–0.80	0.003	0.934
*Box–Cox transformed variables*					
*Age, years*	Transformation not applied	Transformation not applied	Transformation not applied	−0.574	<0.001
*AMH_BC*	−0.06 ± 1.48	0.07 (−1.06–1.04)	−2.42–3.00	Reference	—
*CRP_BC*	1.27 ± 1.76	1.04 (0.71–2.43)	−2.14–7.09	−0.033	0.337
*Neutrophil_BC*	1.41 ± 0.43	1.38 (1.14–1.71)	−0.05–2.59	0.065	0.059
*Lymphocyte_BC*	0.97 ± 0.64	0.98 (0.59–1.38)	−0.99–3.64	0.024	0.480
*NLR_BC*	0.57 ± 0.40	0.55 (0.33–0.77)	−1.17–1.68	0.042	0.224

Note: Correlation coefficients for raw variables were calculated against raw AMH values. Correlation coefficients for Box–Cox transformed variables were calculated against Box–Cox transformed AMH values (AMH_BC). Pearson correlation analysis was used. AMH, anti-Müllerian hormone; CRP, C-reactive protein; NLR, neutrophil-to-lymphocyte ratio; NRBC, nucleated red blood cell; SD, standard deviation; Q1, 25th percentile; Q3, 75th percentile; BC, Box–Cox transformed.

**Table 2:** Comparison of alternative Tobit regression models according to multicollinearity, model fit, and predictive performance.

Model Characteristic	Model 1: Component Model	Model 2: Ratio Model	Model 3: Full Model	Model 4: Neutrophil Model
Variables Included	Age, CRP_BC, Neutrophil_BC, Lymphocyte_BC	Age, CRP_BC, NLR_BC	Age, CRP_BC, Neutrophil_BC, Lymphocyte_BC, NLR_BC	Age, CRP_BC, Neutrophil_BC
**Variance inflation factors**				
Age	1.016	1.000	1.007	1.016
CRP_BC	1.039	1.000	1.001	1.017
Neutrophil_BC	1.055	—	6.919	1.002
Lymphocyte_BC	1.050	—	6.914	—
NLR_BC	—	1.000	Not estimable due to collinearity	—
**Model fit indices**				
Log-likelihood	−1364.78	−1364.95	−1364.08	−1364.96
AIC	2741.56	**2739.90**	2742.16	2739.92
BIC	2770.02	**2763.62**	2775.36	2763.64
Original-scale R^2^	0.2288	0.2288	Not evaluated because of multicollinearity	0.2288
RMSE, ng/mL	1.7439	1.7439	Not evaluated because of multicollinearity	1.7439
MAE, ng/mL	1.1114	**1.1114**	Not evaluated because of multicollinearity	1.1115

Note: Model 2 was selected as the final model because it provided the lowest AIC and BIC values while avoiding multicollinearity and retaining a clinically interpretable inflammatory ratio variable. Model 3 was not considered for final interpretation because simultaneous inclusion of neutrophil count, lymphocyte count, and NLR resulted in problematic multicollinearity. AIC, Akaike information criterion; BIC, Bayesian information criterion; CRP, C-reactive protein; MAE, mean absolute error; NLR, neutrophil-to-lymphocyte ratio; RMSE, root mean squared error; BC, Box–Cox transformed.

**Table 3:** Age-adjusted associations of CRP and NLR with Box–Cox-transformed AMH in the final Tobit regression model.

*Predictor*	*β Coefficient*	*95% CI*	*p Value*
*Age, years*	−0.123	−0.135 to −0.111	<0.001
*CRP_BC*	−0.038	−0.085 to 0.009	0.114
*NLR_BC*	−0.012	−0.222 to 0.198	0.912

Note: The dependent variable was Box–Cox-transformed AMH. Coefficients and 95% confidence intervals are presented on the transformed scale. The intercept and scale parameter were omitted because the table focuses on the clinically relevant predictor estimates. AMH, anti-Müllerian hormone; CRP, C-reactive protein; NLR, neutrophil-to-lymphocyte ratio; BC, Box–Cox transformed.

**Table 4:** Predictive performance of alternative modeling approaches after back-transformation to the original AMH scale.

*Predictive Performance Metric*	*Log-Tobit Model*	*Box–Cox OLS Model*	*Final Box–Cox Tobit Model*
*Original-scale R^2^*	0.1477	0.2288	0.2288
*RMSE, ng/mL*	2.0081	1.7439	1.7439
*MAE, ng/mL*	1.2043	1.1114	1.1114

Note: Predictive performance was assessed after back-transformation of model predictions to the original AMH scale in ng/mL. The final Box–Cox Tobit model was selected because it accounted for both the skewed distribution of AMH and the left-censoring caused by the assay’s lower limit of detection. AMH, anti-Müllerian hormone; MAE, mean absolute error; OLS, ordinary least squares; RMSE, root mean squared error.

## Data Availability

The original contributions presented in this study are included in the article. Further inquiries can be directed to the corresponding author.
